# Cytokine Profiling in Different SARS-CoV-2 Genetic Variants

**DOI:** 10.3390/ijms232214146

**Published:** 2022-11-16

**Authors:** Zoia R. Korobova, Natalia A. Arsentieva, Natalia E. Liubimova, Oleg K. Batsunov, Vladimir G. Dedkov, Anna S. Gladkikh, Alena A. Sharova, Zhansaya Adish, Ekaterina I. Chernykh, Victor A. Kaschenko, Vyacheslav A. Ratnikov, Victor P. Gorelov, Oksana V. Stanevich, Alexandr N. Kulikov, Dmitry E. Pevtsov, Areg A. Totolian

**Affiliations:** 1 Saint Petersburg Pasteur Institute, 14 Ulitsa Mira, 197101 Saint Petersburg, Russia; 2 Intensive Care Unit, Department of Immunology, Department of Infectious Diseases, Pavlov First State Medical University of St. Petersburg, 6–8 Ulitsa L’va Tolstovo, 197022 Saint Petersburg, Russia; 3 Laboratory of Immunochemistry and Immunobiotechnology, National Center for Biotechnology, 13/5, Kurgalzhynskoye Road, Nur-Sultan 010000, Kazakhstan; 4 The Federal State Budgetary Institution ‘North-Western District Scientific and Clinical Center Named after L.G. Sokolov Federal Medical and Biological Agency’, Prospekt Kul’tury, 4, 194291 Saint Petersburg, Russia; 5 Department of Faculty Surgery, Saint Petersburg State University, Universitetskaya Naberezhnaya, 7/9, 199034 Saint Petersburg, Russia; 6 Scientific, Clinical and Educational Center “Radiation Diagnostics and Nuclear Medicine” of the Institute of High Medical Technologies, Saint Petersburg State University, Universitetskaya Naberezhnaya, 7/9, 199034 Saint Petersburg, Russia

**Keywords:** COVID-19, cytokines, multiplex analysis, variants of concern

## Abstract

This study is a successor of our previous work concerning changes in the chemokine profile in infection that are associated with different SARS-CoV-2 genetic variants. The goal of our study was to take into account both the virus and the host immune system by assessing concentrations of cytokines in patients infected with different SARS-CoV-2 variants (ancestral Wuhan strain, Alpha, Delta and Omicron). Our study was performed on 340 biological samples taken from COVID-19 patients and healthy donors in the timespan between May 2020 and April 2022. We performed genotyping of the virus in nasopharyngeal swabs, which was followed by assessment of cytokines’ concentration in blood plasma. We noted that out of nearly 30 cytokines, only four showed stable elevation independently of the variant (IL-6, IL-10, IL-18 and IL-27), and we believe them to be ‘constant’ markers for COVID-19 infection. Cytokines that were studied as potential biomarkers lose their diagnostic value as the virus evolves, and the specter of potential targets for predictive models is narrowing. So far, only four cytokines (IL-6, IL-10, IL-18, and IL-27) showed a consistent rise in concentrations independently of the genetic variant of the virus. Although we believe our findings to be of scientific interest, we still consider them inconclusive; further investigation and comparison of immune responses to different variants of SARS-CoV-2 is required.

## 1. Introduction

In the timespan of nearly 3 years, the COVID-19 pandemic has claimed over 6 million human lives [1]. Caused by a single-stranded RNA virus, this infectious disease usually affects both upper and lower respiratory tracts. It provides a wide spectrum of symptoms, and it varies in severity for each individual: from common cold to pneumonia with respiratory distress syndrome [2]. Such a variability in disease manifestation and its severity is still yet to be explained.

One of the possible explanations lies within the main principle of epidemiology that the severity of any infectious disease is based on the three key factors: environment, the infectious agent and the host [3]. While the involvement of environmental factors is too complex to assess in COVID-19, the viral properties and reactivity of the host immune system are more available for retrospective study on clinical material.

Before addressing the infectious agent within the COVID-19 pandemic, one must take into consideration that the virus itself is constantly evolving. The SARS-CoV-2 virion has changed significantly since the emergence of the virus in the human population, creating a distinctive group of so-called ‘variants of concern’ (VoC). According to the World Health Organization (WHO), changes in the genetic structure of the virus affect its properties, such as transmissibility and virulence. Thus, modifications of the virion are altering the clinical course of the disease and making public health measures to prevent viral transmission less effective [4]. With the emergence of more VoCs over the years, it is now impossible to characterize the clinical presentation of COVID-19 without taking into consideration differences made by mutations in the viral genome.

As for the host, one of the most prominent factors in the infectious process is the host immune system. It is deeply involved in the development of infection, and COVID-19 is not an exception in that matter. For instance, when invading the host, the SARS-CoV-2 virion avoids immune responses and causes the pathological activation of cytokines, which is followed by the so-called ‘cytokine storm’ [5]. Such a phenomenon is usually recognized as one of the death causes in COVID-19 [6]. Studying the cytokine profile as one of the markers of host immune reactivity has already proven to be an important area of research when exploring SARS-CoV-2-associated infection [5,6,7,8,9].

The goal of our study was to take into account both the virus and the host immune system by assessing concentrations of cytokines in patients infected with different SARS-CoV-2 variants (ancestral Wuhan strain, Alpha, Delta and Omicron).

## 2. Results

For cytokines concentrations, we decided to make comparisons not only between cohorts of different SARS-CoV-2 genetic variant but also of different severity. This allowed us to investigate each variant separately. For convenience, we separated cytokines into families based on receptors.

### 2.1. Cytokines of Type I Receptor Family

When comparing concentrations of major ligands (IL-2, IL-3, IL-4, IL-5, IL-6, IL-7, IL-9, IL-12 (p40), IL-12 (p70), IL-15, IL-27, GM-CSF, G-CSF) to type I cytokine receptors family between COVID-19 patients and healthy donors, we noted statistically significant elevation of IL-3 and IL-5 for the Wuhan strain, IL-6 for all the variants, but more so for the severe Alpha variant; IL-12 (p40) for the Wuhan strain and the Alpha variant. For IL-27, however, concentrations in patients with acute COVID-19 were significantly higher independently of the variant. G-CSF showed an increase for patients with the Wuhan strain and Alpha variant. Interestingly, GM-CSF was increased only in samples from patients with the Wuhan strain, whereas for all the other variants, it was barely detectable in patients’ plasma. IL-7 showed elevation in concentrations for the Wuhan strain, whereas IL-5 showed a prominent increase in both the original Wuhan strain and the Alpha variant.

A comparison of cohorts infected with the same variants but of different severity showed statistically significant differences for IL-6 (the Alpha variant) and IL-18 (the Wuhan strain).

For IL-2, IL-9 and IL-12 (p70), we did not see statistically significant differences in concentrations compared to healthy donors.

**Figure 1:**
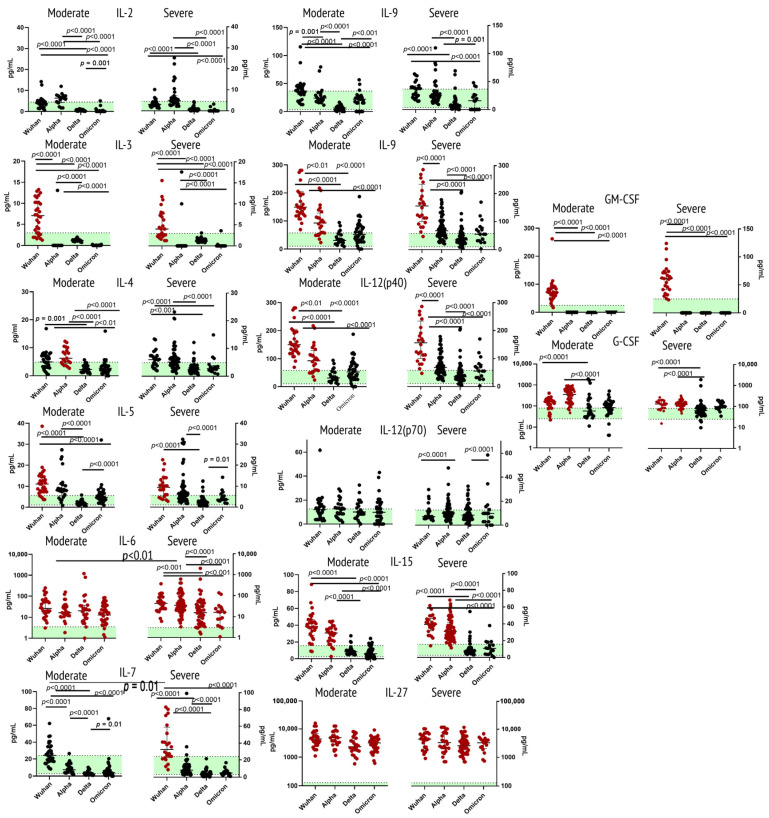
Concentrations of type I receptor family (IL-2, IL-3, IL-4, IL-5, IL-6, IL-7, IL-9, IL-12 (p40), IL-12 (p70), IL-15, IL-27, GM-CSF, G-CSF) in plasma samples from patients infected with different variants of SARS-CoV-2: the original Wuhan strain (*n* = 59), the Alpha variant (*n* = 95), the Delta variant (*n* = 78) or the Omicron variant (*n* = 57). Note: Cytokine comparison was performed based on disease severity (moderate, severe) of COVID-19. The green horizontal stripe represents interquartile range (Q25–Q75) for healthy donors (*n =* 51). The color red highlights groups that show statistically significant differences in comparison with healthy donors. Comparative analysis was performed with one-way ANOVA.

Figure 1 illustrates findings for this group of cytokines.

### 2.2. Cytokines of Type II Receptor Family & TNF Receptor Superfamily

Within the type II receptor family, we analyzed concentrations of IL-10, IL-22, IFNα2 and IFNγ. IL-10 concentrations were significantly higher than in healthy donors for all cohorts, except for the moderate Omicron. When comparing this cytokine in the blood samples of patients infected with the Omicron of different severity, we noted a statistically significant difference. For IL-22, concentrations in most variants, except for the Wuhan strain, witnessed a statistically significant decrease in comparison with healthy donors. Levels of plasma IFNα2 showed an increase in patients infected with the Alpha variant. For IFNγ, however, such an increase was present in samples from patients infected with the Wuhan strain.

TNFα levels were significantly higher in patients with the ancestral Wuhan strain and the Alpha variant when compared with healthy donors and other variants. For TNFβ, elevation of concentrations was noted in samples from patients infected with the Wuhan strain.

**Figure 2:**
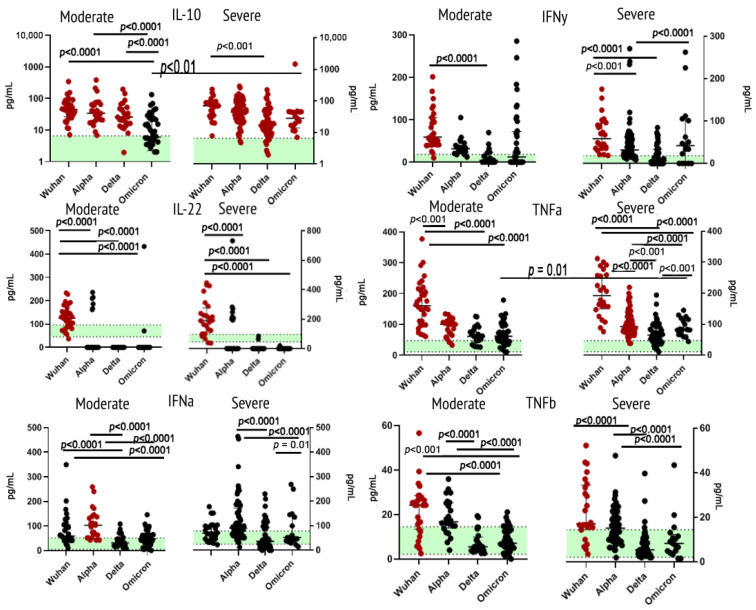
Concentrations of type II receptor family (IL-10, IL-22, IFNα and IFNγ; TNFα, TNFβ) in plasma samples from patients infected with different variants of SARS-CoV-2: the original Wuhan strain (*n* = 59), the Alpha variant (*n* = 95), the Delta variant (*n* = 78) or the Omicron variant (*n* = 57).

Figure 2 illustrates findings within this group of cytokines.

### 2.3. Cytokines of Immunoglobulin Superfamily

Within ligands to immunoglobulin superfamily of receptors, we analyzed concentrations of the following cytokines/growth factors: IL-1α, IL-1β, IL-1RA, IL-18, M-CSF, PDGF-AA, PDGF-AA/BB. IL-1β concentrations were significantly higher in samples from Wuhan strain-infected individuals, whereas for other variants of SARS-CoV-2, the concentrations stayed mostly within norms. When comparing concentrations of IL-1RA between variants, we noted significant elevation of this cytokine compared to healthy donors in all cohorts, except for those who were infected with the Omicron variant. The concentration of IL-1RA did not show significant differences depending on severity. The cohort of patients infected with the ancestral Wuhan strain showed the highest levels of this cytokine. For IL-18, the highest concentrations were detected in samples from patients with severe Wuhan strain viral infection. M-CSF showed a statistically significant rise of concentrations in samples from patients infected with the Wuhan or Alpha variant. For PDGF-AA and PDGF-AB/BB, we noted not only an elevation of these cytokines in samples from the Wuhan strain cohort but also a statistically significant decrease for the Alpha and the Delta variants in comparison with the healthy donors.

IL-1α, while showing no statistically significant differences in comparison with healthy donors, still showed the highest concentrations in patients infected with the Wuhan or Alpha variant.

**Figure 3:**
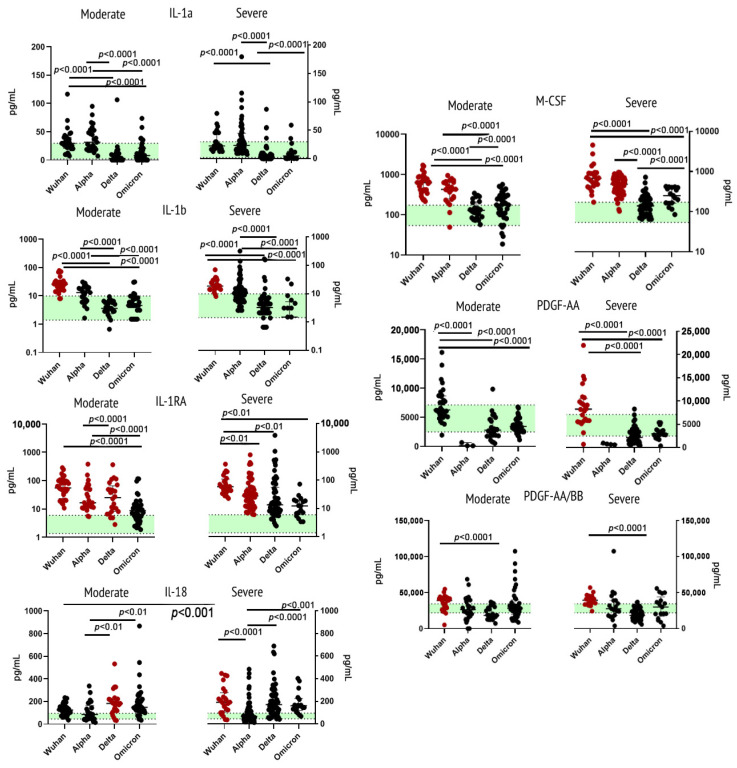
Concentrations of immunoglobulin superfamily cytokines/growth factors (IL-1α, IL-1β, IL-1RA, IL-18, M-CSF, PDGF-AA, PDGF-AA/BB) in plasma samples from patients infected with different variants of SARS-CoV-2: the original Wuhan strain (*n* = 59), the Alpha variant (*n* = 95), the Delta variant (*n* = 78), or the Omicron variant (*n* = 57). See notes for Figure 1.

Figure 3 illustrates findings for this group of cytokines/growth factors.

### 2.4. Cytokines/Growth Factors of Other Receptor Families

Measurements of levels of IL-17A, EGF, FGF-2/FGF-basic, Flt3 Ligand, TGF-α, and VEGF-A in blood plasma showed the most prominent increase in concentrations of the cytokines in patients infected with the original Wuhan strain or the Alpha variant.

For instance, EGF showed a statistically significant increase in concentrations for the Wuhan cohort in comparison with healthy donors and patients infected with other variants of SARS-CoV-2. For FGF-2/FGF-basic, the upsurge of concentration was noted in the moderate Alpha and severe Wuhan variants. The FLT3 ligand showed increased levels in patients infected with the Alpha variant and in samples from the severe course of the Wuhan strain. For VEGF-A, plasma levels were the highest in samples from patients infected with the Wuhan strain. Within this cohort, VEGF-A levels were higher in severe COVID-19.

Two cytokines showed no statistically significant differences in comparison to healthy donors: IL-17 and TGFα. For IL-17A, the Wuhan strain showed the highest concentrations. As for TGFα, concentrations of this growth factor in plasma of COVID-19 patients with the Alpha variant were higher than in other variants.

**Figure 4:**
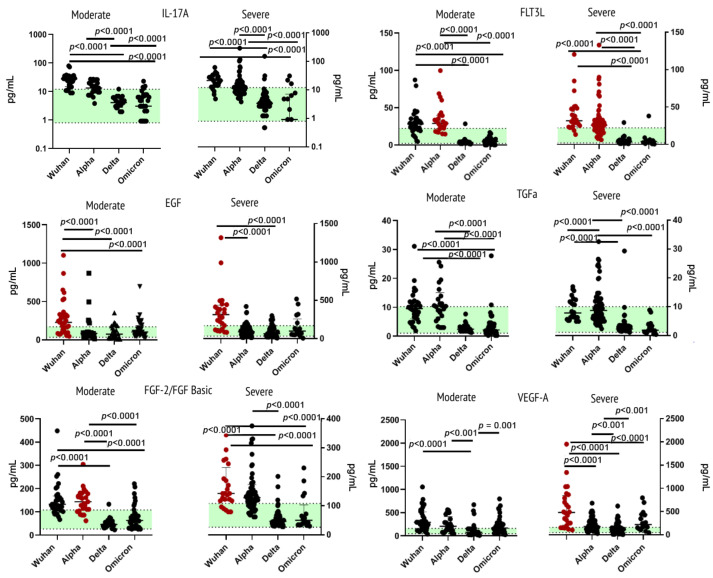
Concentrations of growth factors (IL-17A, EGF, FGF-2/FGF-basic, Flt3 Ligand, TGF-α and VEGF-A) in plasma samples from patients infected with different variants of SARS-CoV-2: the original Wuhan strain (*n* = 59), the Alpha variant (*n* = 95), the Delta variant (*n* = 78) or the Omicron variant (*n* = 57). See notes for Figure 1.

Elaborated information on growth factors is presented in Figure 4.

## 3. Discussion

A general comparison between variants showed significantly more prominent hypercytokinemia in patients infected with the original Wuhan strain or the Alpha variant of SARS-CoV-2. We discussed hypercytokinemia in terms of the Wuhan wild strain in the beginning of the pandemic [10]. However, now, we see that the question of COVID-19 clinical course and cytokine profile is still relevant as new variants emerge. The Delta and the Omicron variant showed lesser influence on the cytokine profile of patients with moderate infection; however, for patients with severe infection, the cytokine profile was more distinguished. Such a finding can be explained by clinical differences between the presentation of SARS-CoV-2 genetic variants: for instance, while the original Wuhan strain showed a tendency to affect the lower respiratory tract and lung tissue, patients with Delta and Omicron tend to present with symptoms of running nose and sneezing more often [11]. Although the Delta variant is often recognized as the variant with higher morbidity and mortality [12], in our study, this variant showed milder cytokine release than other variants. This notion might be explained by the idea of the Delta variant being more successful in avoidance of immune responses in contrast to earlier variants [13]. The following emergence of the Omicron variant [14] might be explained by similar ideas.

**Figure 5:**
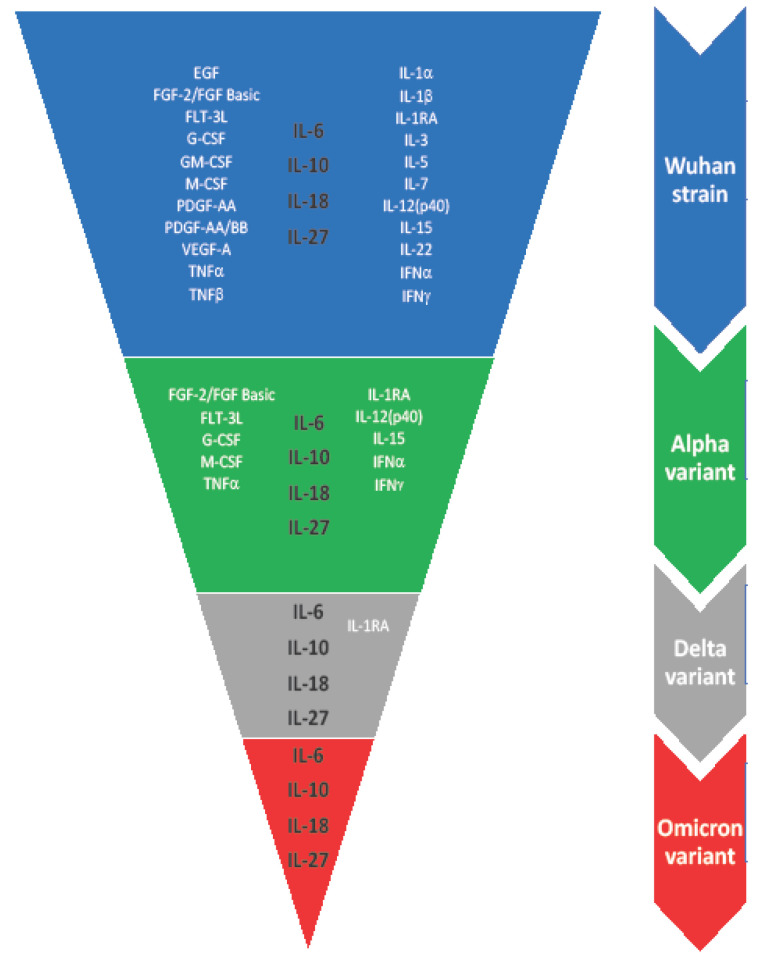
Spectrum of cytokines showing statistically significant (*p* < 0.01) changes in comparison with healthy donors cohort (*n =* 51). Each color represents one of the four variants of concern of SARS-CoV-2 (blue for the original Wuhan strain, green for the Alpha variant, gray for the Delta variant, and red for the Omicron variant).

Although cytokines are often addressed as potential markers for building predictive models for COVID-19 clinical course and outcomes, our study shows that there are still questions to answer. Differences in the cytokine profile of four different SARS-CoV-2 variants are presented in Figure 5. The spectrum of potential markers is narrowing as the virus evolves.

### 3.1. Cytokines of Type I Receptor Family

#### 3.1.1. Interleukin 2

Interleukin 2 (IL-2) plays a major role in orchestrating mechanisms of adaptive immunity: not only does it ensure the maturation of naive T cells, but it also stimulates the differentiation of cytotoxic T lymphocytes [15]. In our study, levels of IL-2 in the blood plasma of COVID-infected patients with moderate and severe infection did not differ from concentrations of this cytokine in healthy donors. Such findings are common in studies comparing COVID-19 patients to healthy donors [16,17]. However, the Alpha variant showed an increase in IL-2 in comparison with other variants, whilst concentrations in the Delta variant cohort were lower. A decrease in IL-2 concentrations in Delta variant is yet to be explained. We can only speculate that such a decline is caused by either the depletion or dysregulation of CD4+ T-cells. We will address that issue in future studies.

#### 3.1.2. Interleukin 3

Interleukin 3 (IL-3) is mostly produced by T cells [18], its main role being stimulation of the myelomonocytic cells, providing the activation of macrophages and granulocytes. The spectrum of cells activated by IL-3 is so wide that originally, this cytokine was referred to as ‘multi colony-stimulating factor’ (multi-CSF) [19]. Within our study, concentrations of this interleukin varied depending on the variant. The original Wuhan strain showed a statistically significant increase in concentrations. During the first wave of the COVID-19 pandemic, IL-3 was suggested as one of the potential markers of severity [20]. However, when comparing samples from first-wave patients infected with this variant, we did not observe any statistically significant distinctions between groups of different severity. When searching for studies of this cytokine concentrations for other variants, we did not see any findings concerning its predictive potential. Our findings on IL-3 in other variants are inconclusive: its concentrations varied from barely detectable to early 20 pg/mL.

#### 3.1.3. Interleukin 4

In cellular immune responses, interleukin 4 (IL-4) controls the differentiation of naive T cells to T helpers, whereas its role in humoral responses is to provide a subclass switch to IgG1 [21]. Our study showed that the concentrations of this cytokine in patients with COVID-19 did not differ from healthy donors, allowing us to presume that the SARS-CoV-2 virion life cycle does not interfere with cellular responses to infection. However, IL-4 is shown to be associated with the remodeling of lung tissue in the late stages of pneumonia [22], whereas our patients were recruited in the study on earlier stages of disease. Moreover, our previous study showed that out of all IgG subclasses, IgG3 is prevalent in earlier stages of the infection, whereas IgG1 levels peak from days 20 to 30 [23]. Therefore, it may be speculated that the involvement of IL-4 is expected as disease progresses.

#### 3.1.4. Interleukin 5

Interleukin 5 (IL-5) is known not only as one of the potential activators of B cellular responses but as the major factor for eosinophils’ maturation [24]. Despite the original idea of eosinophilic inflammation (e.g., in asthma) being an additional factor to disease severity, it is now proven that higher eosinophils levels negatively correlate with disease severity in COVID-19 [25]. Our study showed a prominent increase in IL-5 concentrations in patients with the Wuhan variant, whereas in the Delta variant, IL-5 levels were lower in comparison with other variants. Eosinophilic count is also tied to CCL11/Eotaxin, which is a chemokine that controls eosinophils chemoattraction. Previously, we already studied CCL11/Eotaxin along with other chemokines in different COVID-19 genetic variants [26]. Interestingly enough, it showed a similar picture as IL-5.

#### 3.1.5. Interleukin 6

Interleukin 6 (IL-6) is one of the most recognized factors of the inflammatory responses; its role in COVID-19 has been extensively studied in recent years. Interleukin 6 promotes cell differentiation, and it induces the synthesis of acute phase proteins [27]. Its concentrations usually serve as biological markers of cytokine storm, rising as disease progresses [28]. As we predicted, our study showed a significant increase in concentrations of IL-6 independently of the variant. Previously, we highlighted an elevation of IL-6 as one of the COVID-19 characteristic features [29].

#### 3.1.6. Interleukin 7

Interleukin 7 (IL-7) is an important mediator of T and B-cellular differentiation; it is involved in thymopoesis and balances T cell apoptosis and survival [30]. In COVID-19, the role of IL-7 is usually investigated through the prism of additional assessment of cellular responses (CD4+ and CD8+ lymphocytes) [31]. As with several previous cytokines, the Wuhan strain showed elevated concentrations of IL-7 compared not only to healthy donors but to other genetic variants. In the early pandemic, this cytokine was described as yet another marker of disease severity [32,33]. However, we did not find any data concerning other genetic variants of SARS-CoV-2. Within our work, IL-7 concentrations in samples from other cohorts did not differ from healthy donors.

#### 3.1.7. Interleukin 9

Interleukin 9 (IL-9) is functionally characterized as a growth factor for repetitively stimulated T cell lines but not for naive T cells; it is also active in relation to mast cells [34]. In our study, this cytokine showed little involvement in the inflammatory process; its concentrations did not differ from healthy donors independently of the variant. This correlates with the findings in other studies [35]. Research of the literature on the role of IL-9 in COVID-19 showed more interest in IL-9 producing cells and not in the cytokine itself [36]. However, in patients infected with the Wuhan strain and presenting severe course of the disease, concentrations of IL-9 were higher than in other variants.

#### 3.1.8. Interleukin 12 (p40 & p70)

Interleukin 12 (IL-12) is a multifunctional cytokine; it consists of two subunits (p35 and p40) required to produce a bioactive form of this interleukin, p70 [37]. In COVID-19, concentrations of plasma IL-12 is often lower in patients with severe infection [16]. IL-12 (p70) in association with other factors, such as arterial hypertension and anti-inflammatory cytokine IL-10, has shown to be the predictive marker of COVID-19 severity [38]. In our study, plasma concentrations of p40 subunits showed higher concentrations in the Wuhan and Alpha variant; however, the bioactive form of IL-12 stayed within interquartile ranges of IL-12 (p70) concentrations in healthy donors. Some studies show an antagonistic function of IL-12 (p40) in relation to IL-12 (p70) [39]; therefore, such a finding is not surprising.

#### 3.1.9. Interleukin 15

Interleukin 15 (IL-15) is an important mediator of innate immunity, as it ensures the differentiation of natural killer (NK) cells. In addition to its crucial part in the innate immunity, IL-15 modulates the formation of T memory cells, which are agents of adaptive immunity [40]. In our study, the elevation of IL-15 concentration was noted for the Wuhan and the Alpha cohort. An extensive search of the literature concerning IL-15 in COVID-19 development showed little data; however, we found that IL-15 is one of the first cytokines to increase its concentration in response to mRNA vaccination [41]. It is possible that the increase in IL-15 is associated with the early innate responses and the development of immunological memory to the SARS-CoV-2 antigenic structure.

#### 3.1.10. Interleukin 27

Interleukin 27 (IL-27) is related to IL-12, as IL-27 synergizes with IL-12 to promote IFNγ production by CD4, CD8 T cells and NKT cells. IL-27 is an early initiator of Th1 differentiation, and it is an inhibitor of Th17 cells [42]. This cytokine and its role in COVID-19 has previously been under the scope of our research: we investigated its association with T cellular subsets, as it negatively correlated with the CCR6+ cells in acute COVID-19 patients with the Wuhan variant [43]. Within the current study, we noted a significant elevation in concentrations of this cytokine independently of the variant. We presume that concentrations of IL-27 can serve as the marker of intercellular activation as well as of an increase in cytolytic activity directed at virus-subdued cells.

#### 3.1.11. G-CSF and GM-CSF

Granulocyte-macrophage colony-stimulating factor (GM-CSF) and granulocyte colony-stimulating factors (G-CSF) are hemopoetic growth factors that can stimulate the growth of cells both locally and in the paracrine fashion; they are known to be involved in host responses against infections; in heathy donors, its secretion is low [44,45]. In COVID-19-centered studies, both these factors are mostly discussed as a therapeutic option in the form of the GM-CSF drug. Our study showed an increase in GM-CSF concentrations in patients with the original Wuhan strain; for other variants, its concentrations were below detection limits. One of the studies comparing concentrations of cytokines between different waves of COVID-19 highlighted that GM-CSF concentrations lost their significance as a severity marker as time progressed [46]. G-CSF concentrations were the highest in the Wuhan and the Alpha cohorts; in Delta and Omicron cohorts, G-CSF concentrations did not exceed the interquartile range of healthy donors’ G-CSF concentrations.

### 3.2. Cytokines of Type II Receptor Family and TNF Receptor Superfamily

#### 3.2.1. Interleukin 10

Interleukin 10 (IL-10) is a so-called ‘anti-inflammatory’ cytokine, usually preserving the development of autoimmune reaction after the effective clearance of infectious agents [47]. In COVID-19, the early stages of disease are characterized by the dramatic rise in concentrations of this cytokine [48]; our work is no different as concentrations of IL-10 were higher in severe COVID-19. Moreover, moderate Omicron showed lower concentrations compared to other variants of the same severity—a notion that can potentially be explained by clinical features of this specific variant.

#### 3.2.2. Interleukin 22

Interleukin 22 (IL-22) is a cytokine with pleiotropic function; it affects not only hematopoietic cells but also non-hematopoietic cells (e.g., epithelial cells and fibroblasts). It is abundantly present in the barrier tissues of the host [49]. IL-22 plays a prominent role as the mediator for protective immunity; its specific function as a protector of the lung tissue has been underlined in COVID-related studies [50,51].

In our study, we noted a statistically significant elevation of this cytokine in patients with the Wuhan variant; however, in most samples from patients infected with other variants, concentrations of this cytokine were below detectable limits.

#### 3.2.3. Interferons α and γ

Cytokines of the interferon (IFN) family are a significant part of COVID-associated inflammation: the dysregulation of IFNγ signaling by SARS-CoV-2 leads to hyperinflammatory responses in the lung [52]. In our study, the imbalance of IFN production was evident: while type I IFNα in our study showed an increase in concentrations in patients with the original Wuhan strain, other variants showed little differences from healthy donors. However, levels of type III IFNγ were higher than concentrations of this cytokine in healthy donors. These findings put an important question of timing: how does IFNγ secretion change as disease progresses? Due to the constant shift in IFNγ dynamic (inhibition vs. hyperinflammatory profile), it is difficult to differentiate the influence of the genetic variant and the timeline on IFN concentrations. It is, however, evident that the antigenic structure of the virus plays a certain role in IFNγ production in the host.

#### 3.2.4. Tumor Necrosis Factors α and β

Members of the tumor necrosis factors (TNF) family control numerous immune system functions, orchestrating inflammatory responses via the induction of cytokine production and cellular co-stimulation [53]. The rise of concentrations of TNFα, witnessed within our study, is a common finding in COVID-19 patients; this pro-inflammatory cytokine is often designated with the role of severity predictor [54,55]. TNFβ (also known as Lymphotoxin α) is usually involved in autoimmune processes; it play a pivotal role in the development of rheumatoid arthritis [56]. Our investigation showed a statistically significant increase in this factor in the Wuhan strain cohort; there was also an increase in patients infected with the Alpha variant. However, for other variants (i.e., Delta and Omicron), concentrations of the above-mentioned cytokine did not differ from those in healthy donors. Even though COVID-19 is often followed by autoimmune complications, its antigenic structure is an important factor affecting the severity and course of such complications [57]. Therefore, differences in genetic variants of the virion might be able to affect the development and manifestation of autoimmune reactions, including those mediated by TNFβ.

### 3.3. Cytokines of Immunoglobulin Superfamily

#### 3.3.1. Interleukin 1 (α and β) and Its Receptor Antagonist

Interleukin 1 (IL-1) is composed of two cytokines, IL-1α and IL-1β, and it is considered to be a pro-inflammatory cytokine; its secretion is mostly induced at early responses by innate immunity cells [58]. Both IL-1α and IL-1β are reportedly correlating with disease severity [59]; however, our study showed a very weak elevation of both cytokines in the plasma of infected donors. Predictably, concentrations of IL-1 within our study showed a more prominent increase in patients infected with the Wuhan strain, potentially marking hypercytokinemia in the first wave of the pandemic to be more eminent in patients.

On the contrary, interleukin 1 receptor antagonist (RA) is the anti-inflammatory opposition of IL-1. By binding to the IL-1 receptor non-productively, this cytokine blocks signaling of the IL-1 pathway, bringing balance to immune responses to infection and vaccination [60]. Interestingly, IL-1RA concentrations were elevated not only in the Wuhan and the Alpha strain but also in the Delta and the Omicron cohorts.

#### 3.3.2. Interleukin 18

Interleukin 18 (IL-18) is involved in multiple immune processes within the host, and its role is mostly protective; alongside IL-12, it participates in IFNγ production. In addition to immunoregulatory function, IL-12 shows properties of pro-inflammatory cytokines [61]. When comparing IL-18 concentrations in diseased patients and healthy donors, we noted an increase in all four variants. Interestingly, the Delta variant showed elevation of this interleukin as high as was observed in the Wuhan strain cohort. The question of anti-inflammatory responses in the body on different stages of COVID-19 infection is as important as the question of different clinical presentation between VoC.

#### 3.3.3. Macrophage Colony-Stimulating Factor

Macrophage colony-stimulation factor (M-CSF) is constitutively expressed in body cells without previous activation [62], its concentrations are detectable even for healthy donors, as shown in our study. According to other researchers during COVID-19, M-CSF levels rise in comparison not only to healthy donors but also to asymptomatic infection [63]; the design of our study did not include asymptomatic donors, but we noted M-CSF elevations in infected cohorts nonetheless. Numbers for the Wuhan strain and the Alpha variant witnessed the most prominent increase; in the Wuhan variant, the severity was an additional factor affecting this cytokine.

#### 3.3.4. Platelet-Derived Growth Factor (AA and AA/BB)

Platelet-derived growth factor is a dimeric glycoprotein that can be composed of two A subunits (PDGF-AA), two B subunits (PDGF-BB), or one of each (PDGF-AA/BB); its functional activity is mostly directed at angiogenesis, but it potentially might affect fibroblasts and other non- hematopoietic cells [64]. Our results on both PDGF-AA and PDGF-AA/BB showed controversial results. While the original Wuhan strain showed an increase in both factors, the Alpha variant showed a drop in concentrations compared to healthy donors. Our colleagues received similar results: the concentrations of PDGF-AA and PDGF-AA/BB witness a depletion in samples collected from COVID-19 infected patients in October 2020 [65].

### 3.4. Cytokines/Growth Factors of Other Receptor Families

#### 3.4.1. Interleukin-17A

Interleukin-17 (IL-17) family includes IL-17A, IL-17B, IL-17C, IL-17D, IL-17E and IL-17F. This cytokine is essential for Th-17 functional activity, it is known to show slightly increased plasma and serum levels in patients with COVID-19 with little relation to severity [66]. In our study, however, the rise of concentrations was noted exclusively in the Wuhan strain cohort. Other variants show little to no difference compared to healthy donors.

#### 3.4.2. Epidermal Growth Factor

Epidermal growth factor (EGF) is a protein-structured growth factor that stimulates cell division, differentiation, survival, proliferation, growth, and cell migration. Its receptor is extensively presented in postmortem lung tissue samples from patients with COVID-associated pneumonia [67], as EGF seems to be involved in the formation of lung fibrosis in severe cases. Concentrations of EGF in the blood plasma of patients with COVID-19 showed an increase in this cytokine for the Wuhan strain; other variants show no indication of hypercytokinemia in terms of EGF.

#### 3.4.3. Fibroblast Growth Factor-2

Fibroblast growth factor-2/Basic fibroblast growth factor (FGF-2/FGF-basic) is a growth factor possessing numerous functions and effects on body cells and tissues. Its concentrations are known to rise with the progression of disease severity in COVID-19 [68]. In our study, we noted a statistically significant increase in this growth factor in the Wuhan strain and the Alpha variant; interestingly, the Delta variant did not show any changes in terms of FGF-2 concentrations compared to healthy donors.

#### 3.4.4. Fms-like Tyrosine Kinase 3 Ligand

Fms-like tyrosine kinase 3 ligand (Flt-3L) has a limited effect on the growth of hematopoietic progenitors when used alone, but it is very potent in synergizing with other hematopoietic cytokines, such as IL-3, IL-6, GM-CSF, G-CSF and IL-11 [69]. This factor is essential for dendritic cells development; the Alpha variant within our study showed the most prominent rise of Flt-3L levels among the four variants.

#### 3.4.5. Transforming Growth Factor Alpha

Transforming growth factor alpha (TGFα) is a member of EGF family, and its functional role is similar to that of EGF; it binds to the same receptor [70]. Within our study, TGFα concentrations did not show differences in comparison to healthy donors.

#### 3.4.6. Vascular Endothelial Growth Factor A

Vascular endothelial growth factor A (VEGF-A) VEGF is a vascular factor that displays multiple biological functions under physiological and pathological conditions, including embryonic development, hematopoiesis, vasculogenesis, angiogenesis, vascular permeability, and inflammation [71]. Its concentration in lung tissue leads to the increase in vascular permeability and pulmonary edema [68] and respiratory distress syndrome in COVID-19. The highest concentrations were noted for the Wuhan strain.

### 3.5. Constant Markers of COVID-19 Infection (IL-6, IL-10, IL-18, IL-27)

As mentioned above, levels of four cytokines (IL-6, IL-10, IL-18, IL-27) were consistently elevated in patients with COVID-19 independently of the variant.

While three out of four of these cytokines are prone to pro-inflammatory function, one (IL-10) plays their counterpart by providing anti-inflammatory responses. Its function, however, is more than to simply stop immune responses. Its presence is known to activate CD8+ T-cells, with their later exhaustion and IFNᵧ production [47]. In bacterial infections, it is known to enhance the susceptibility of lung tissue to pathogens [72]; in viral infections, the IL-10 pathway dampens NKT responses [73]. It can be speculated that through its anti-inflammatory features, IL-10 can suppress immunity, possibly playing its role as an accomplice to COVID-19 immunopathogenesis.

IL-6 and IL-18 are well-known markers of COVID-associated immunity, and the IL-6 activation pathway is potentially activated by the presence of IL-18 [74]. Not only do these cytokines ensure the activation of adaptive and innate immunity, but they are also responsible for systemic inflammatory effects: fever, vasodilatation, and acute-phase proteins secretion; their presence magnifies existing inflammatory reactions [73].

IL-27 marks early T-cellular responses, providing the polarization and maturation of such cells [42]; as T cytotoxic lymphocytes are the main effectors against viral infections, IL-27 is an important factor of adaptive immunity.

We specifically highlight the role of the four above-mentioned cytokines (IL-6, IL-10, IL-18 and IL-27), as they seem to be ‘constant’ markers for COVID-19 infection. Their concentrations rise independently of the genetic variant of the virus, potentially marking the dysregulation of immune responses to SARS-CoV-2 infection. This study is a successor for our previous work focused on the chemokine profiling of different SARS-CoV-2 genetic variants [26]; overall, it is becoming more evident that the evolution of the virus brings new challenges to immunology.

## 4. Materials and Methods

Our study was performed on 340 biological samples taken from COVID-19 patients and healthy donors in the timespan between May 2020 and April 2022. The samples (blood plasma) belonged to patients infected with one of the four VoC circulating on the territories of Saint Petersburg, Russia in that time. These variants were: the ancestral Wuhan strain (59 samples collected from patients hospitalized in May 2020), the Alpha variant or B.1.1.7 (95 samples collected from patients hospitalized in late November 2020 to March 2021), the Delta variant or B.1.617.2 (78 samples collected from patients hospitalized between August and November 2021), and the Omicron variant or B.1.1.529 (57 samples collected from patients hospitalized between February and April 2022). As controls, we included samples from healthy donors (*n* = 51) in our study.

Whole blood samples were collected in vacuum tubes with EDTA anticoagulant, which was followed by centrifugation (350 g for 10 min) and plasma extraction. Plasma samples were transferred to cryotubes and frozen at −80 °C prior to analysis.

### 4.1. Genetic Testing

The genotyping of SARS-CoV-2 isolates collected from patients was performed via near-complete genome sequences on the Illumina MiSeq automatic platform (Illumina Inc., San Diego, CA, USA). Nasopharyngeal swabs from COVID-19 patients were studied previously using the COVID-19 Amp RT-qPCR Kit (Saint Petersburg Pasteur Institute, Saint Petersburg, Russia) according to the manufacturer’s recommendations for SARS-CoV-2 detection and concentration assessment.

Swabs were collected in 500 µL of a special transport medium (AmpliSens^®^, Moscow, Russia) or phosphate-buffered saline (pH 7.0) and stored at −20 °C until analysis. Total nucleic acid samples were obtained by extraction and purification using the RIBO-prep DNA/RNA Extraction Kit (AmpliSens^®^, Moscow, Russia) according to the manufacturer’s recommendations. DNA/RNA was eluted with 50 µL of the elution buffer (AmpliSens^®^, Moscow, Russia) and stored at −70 °C until molecular analysis.

Reverse transcription was performed using random hexanucleotide primers and the Reverta-L Kit (AmpliSens^®^, Moscow, Russia) according to the manufacturer instructions; cDNA samples were stored at −70 °C and subsequently used as amplification templates.

Libraries were prepared using the TruSeq Nano DNA Kit (Illumina Inc., San Diego, CA, USA) and the TruSeq DNA CD Indexes Kit (Illumina Inc., San Diego, CA, USA). Quality assessment of the final libraries was carried out on the QIAxcel Advanced capillary system (Qiagen, Hilden, Germany). Sequencing was performed using the Illumina MiSeq System (Illumina Inc., San Diego, CA, USA) with the MiSeq Reagent Kit v3 (600-cycle) (Illumina Inc., San Diego, CA, USA). The quality of Illumina reads was assessed using the FastQC program. Raw reads were filtered with Trimmomatic to remove adapters, low-quality nucleotides, and biased sequences at the ends of reads. Genome assembly was carried out by mapping to the SARS-CoV-2 reference genome (NCBI accession number NC_045512.2) using Bowtie 2. For variant calling and consensus generation, samtools and bcftools software were used. The Nextclade tool was used to assess the quality of assembled sequences and to assign genomes to lineages.

All sequencing was performed retrospectively.

### 4.2. Patients

All the samples (*n* = 289) were taken from patients with PCR-verified COVID-19. All the patients were hospitalized with an official diagnosis of COVID-19 at two hospitals in Saint Petersburg, Russia: a COVID-19-specialized hospital, Pavlov First Saint Petersburg State Medical University, and the North-Western Scientific and Clinical Center named after L.G. Sokolov. Therefore, samples from patients with mild COVID-19 were not present in this study.

The patients were recruited to the study at the time of admission, and peripheral blood taken within the first 7–10 days from the onset of infection. All patients included in the study were above 18, had positive COVID-19 PCR testing and a clinically confirmed diagnosis without known previous SARS-CoV-2 infection and/or vaccination. We excluded from the study patients with previously diagnosed immunodeficiencies, with current co-infections or comorbidities in an active stage, including pregnancy. Patients treated with something other than occasional non-steroid anti-inflammatory drugs prior to hospital admission were also excluded from the study. Therefore, antiviral and steroid drug use was not interfering with cytokine concentrations of the samples.

We also included plasma samples of 51 healthy donors with no prior history of COVID-19. These samples were collected at the start of the pandemic (from January to March 2020).

The age and sex characteristics for each cohort are presented in Table 1.

The study’s protocol was approved by the ethics committee of the Saint Petersburg Pasteur Institute (protocol #67, dated 28 April 2021) in accordance with the Declaration of Helsinki. All participants were informed of our study and willingly signed consent forms.

### 4.3. Disease Severity Assessment

The severity of the disease was assessed by hospitals’ medical staff based on the Guidelines of Russian Ministry of Healthcare. These guidelines are following the WHO guidelines on COVID-19 management and treatment [9], and they include criteria for establishing disease severity, e.g., age, oxygen saturation, chest imaging (e.g., CT), fever, inflammatory blood markers and other clinical and laboratory symptoms.

Between cohorts, disease severity varied depending on the variant (Table 2).

### 4.4. Cytokine Concentrations Measurement

For cytokine concentrations assessment, we used xMAP multiplexing technology for Luminex MagPix (Austin, TX, USA). For our study, we used the Millipore^®^ kit (Burlington, MA, USA) to analyze concentrations of the following cytokines and growth factors: IL-1α, IL-1β, IL-2, IL-3, IL-4, IL-5, IL-6, IL-7, IL-9, IL-12 (p40), IL-12 (p70), IL-13, IL-15, IL-17A/CTLA8, IL-18, IL-22, IL-27, IFNα2, IFNγ, TNFα, TNFβ/Lymphotoxin-α (LTA), IL-1ra, IL-10, EGF, FGF-2/FGF-basic, Flt3 Ligand, G-CSF, M-CSF, GM-CSF, PDGF-AA, PDGF-AB/BB, TGF-α, and VEGF-A.

### 4.5. Statistical Analysis

We used a Mann–Whitney U-test to compare continuous variables in two groups. For the comparison of multiple data groups, the Kruskal–Wallis test was used with an additional Dunn’s test for multiple comparisons. When interpreting statistical analysis results, we designated *p* < 0.01 to be statistically significant.

Data analysis was performed in GraphPad Prism 8.0 and the SPSS Statistics version 27 software. Data visualization was conducted via GraphPad Prism 8.0 and Microsoft Excel 2013 graphing tools. Data are presented as medians and 25th and 75th interquartile ranges (Me; Q25–Q75).

## 5. Conclusions

As the antigenic structure of the SARS-CoV-2 virion changes with time, host immune responses undergo alterations. The original version of the virus brought global healthcare to the verge of crisis as intensive care units throughout the world faced an overwhelming wave of patients in critical condition. Extensive data research showed that the search of predictive markers of severity and the outcome has been the priority since the start of the pandemic.

However, with the new variants of concern emerging, the clinical presentation of SARS-CoV-2 infection shifts in new directions, and it inevitably affects molecular interactions of the immune system.

Our study is one of the first to compare cytokine profiles of patients with different variants of SARS-CoV-2, and one of the main concepts behind it is that hypercytokinemia and cytokine storm become less threatening with the emergence of new mutations in the viral genome. Cytokines that were studied as potential biomarkers lose their diagnostic value as the virus evolves, and the specter of potential targets for predictive models is narrowing.

So far, only four cytokines (IL-6, IL-10, IL-18, and IL-27) showed a consistent rise in concentrations independently of the genetic variant of the virus. Although we believe our findings to be of scientific interest, we still consider them to be inconclusive; further investigation and comparison of immune responses to different variants of SARS-CoV-2 is required, as well as building a timeline of cytokine activity throughout the infection.

## Figures and Tables

**Table 1:** Demographical characteristics of patients and healthy donors’ cohort.

Variant		Wuhan (*n =* 59)	Alpha (*n =* 95)	Delta (*n =* 78)	Omicron (*n =* 57)	Healthy Donors (*n =* 51)
Age, years Me (Q25–Q75)		58 (32–75)	72 (28–84)	71 (45–93)	69 (32–910)	49 (24–69)
Gender distribution	Female% (*n*)	66.1% (*n =* 39)	52.7% (*n =* 50)	64.1% (*n =* 50)	61.4% (*n =* 35)	58.8% (*n =* 30)
	Male, % (*n*)	33.3% (*n =* 20)	47.3% (*n =* 45)	35.9% (*n =* 28)	38.6% (*n =* 22)	41.2% (*n =* 21)

**Table 2:** Disease severity in cohorts depending on the SARS-CoV-2 genetic variant.

Variant	Patients, Diagnosed with ‘Moderate’ Infection		Patients, Diagnosed with ‘Severe’ Infection	
	%	*n*	%	*n*
Ancestral Wuhan	54.2	32	45.7	27
Alpha	26.6	35	73.6	60
Delta	34.6	27	65.4	51
Omicron	75.4	43	24.6	14

## Data Availability

Not applicable.
